# Physical Exercise in Myasthenia Gravis: A Systematic Review

**DOI:** 10.3390/healthcare14081100

**Published:** 2026-04-20

**Authors:** Claudia Vinciguerra, Ignazio Leale, Nicasio Rini, Fabio Tiziano Orlando, Liliana Bevilacqua, Paolo Barone, Filippo Brighina, Vincenzo Di Stefano, Giuseppe Battaglia

**Affiliations:** 1 Neurology Unit, Department of Medicine, Surgery and Dentistry “Scuola Medica Salernitana”, University Hospital San Giovanni di Dio e Ruggi d’Aragona, 84131 Salerno, Italy; 2 Sport and Exercise Research Unit, Department of Psychology, Educational Sciences and Human Movement, University of Palermo, 90144 Palermo, Italy; lealeignazio7@gmail.com (I.L.); giuseppe.battaglia@unipa.it (G.B.); 3 Department of Biomedicine, Neuroscience and Advanced Diagnostics (BiND), University of Palermo, 90127 Palermo, Italy; 4 IRCCS SYNLAB-SDN, 80143 Napoli, Italy

**Keywords:** exercise, myasthenia gravis, quality of life, aerobic training, resistance training

## Abstract

**Background:** Myasthenia gravis (MG) is a chronic autoimmune disorder characterized by fluctuating skeletal muscle weakness and fatigue, leading to reduced functional independence and impaired quality of life (QoL). Although exercise has historically been discouraged due to concerns about symptom exacerbation, emerging evidence suggest that structured exercise programs may be safe and beneficial in clinically stable patients. This systematic review critically evaluates current evidence on exercise and physical activity interventions in MG, focusing on effectiveness, safety, and impact on functional outcomes, fatigue, and QoL. **Materials and Methods:** A systematic review was conducted following PRISMA guidelines. Searches were performed in PubMed, Web of Science, Google Scholar, Scopus and ScienceDirect for studies published between 2015 and 2025. Keywords included MG, physical activity, aerobic training, resistance training, and respiratory muscle training. Methodological quality was assessed using the Downs and Black checklist. **Results:** Eight controlled studies met the inclusion criteria, encompassing aerobic, resistance, combined, and respiratory muscle training interventions. Sample sizes ranged from small pilot studies to moderate-size randomized controlled trials. Overall, exercise interventions were well tolerated, with no evidence of sustained symptoms exacerbation. Aerobic and combined programs consistently improved functional capacity, muscle strength, and activities of daily living. Respiratory muscle training demonstrated improvements in pulmonary function and inspiratory muscle strength, although findings were more heterogeneous. Study quality ranged from poor to excellent, with common limitations including small sample size, short follow-up duration, and heterogeneity in exercise programs. **Conclusions:** Current evidence supports the safety and potential efficacy of individualized, symptom-guided exercise interventions in clinically stable MG. Regular physical activity exercise may reduce secondary deconditioning, improve functional outcomes, and enhance QoL. However, larger, high-quality randomized controlled trials with standardized programs and longer follow-up periods are required to strengthen clinical recommendations and clarify long-term effects.

## 1. Introduction

Myasthenia gravis (MG) is a chronic autoimmune disorder of the neuromuscular junction characterized by fluctuating skeletal muscle weakness and fatigue. Despite substantial advances in pharmacological treatments, a considerable proportion of patients continue to experience residual muscle weakness, reduced endurance, and persistent fatigue, even during periods of clinical stability [1,2]. These impairments negatively affect functional independence, participation in daily activities, and health-related quality of life (QoL). Historically, physical activity in MG was approached with considerable caution, due to concerns that exercise could exacerbate muscle weakness or accelerate disease progression. As a result, patients were often advised to avoid strenuous or repetitive activities, which may contribute to physical deconditioning, reduced cardiorespiratory fitness, and further functional decline [1]. Over the past two decades, a growing body of evidence has challenged this paradigm, suggesting that appropriately prescribed and supervised exercise may be both safe and beneficial in clinically stable individuals with MG [2,3]. Recent narrative and scoping reviews have highlighted that aerobic exercise, resistance training, combined multimodal programs, and respiratory muscle training are generally well tolerated in clinically stable MG patients and may improve muscle strength, functional capacity, fatigue, and QoL without increasing the risk of disease exacerbation [1,2,3]. In addition, observational studies indicate that higher levels of habitual physical activity are associated with lower fatigue and better health-related QoL, further supporting the role of physical activity as a potentially modifiable factor in MG management [2,3,4,5,6,7]. However, the available evidence remains limited by small sample sizes, heterogeneity in exercise modalities, intensity, duration, and outcome measures, as well as a predominance of short-term interventions. These limitations underscore the need for a comprehensive synthesis of current data to better inform clinical practice and guide future research [3]. Unlike previous narrative and scoping reviews, this systematic review focuses specifically on controlled interventional studies, providing a more rigorous synthesis of the available evidence on the effectiveness and safety of exercise-based interventions in MG. The aim of this systematic review is therefore to critically evaluate the current evidence on exercise and physical activity interventions in patients with MG, with particular focus on their effectiveness, safety, and impact on functional outcomes, fatigue, and QoL. By synthesizing findings across different exercise modalities and disease subgroups, this review aims to delineate areas of consensus and uncertainty, support evidence-based rehabilitation strategies, and highlight priorities for future research.

## 2. Materials and Methods

### 2.1. Search Strategy

This systematic review was conducted in accordance with the Preferred Reporting Items for Systematic Reviews and Meta-Analyses (PRISMA) guidelines [8]. The review was registered in the PROSPERO International Prospective Register of Systematic Reviews (Registration number: CRD420261323420) and was conducted in accordance with the predefined protocol. A comprehensive literature search was performed in PubMed, Web of Science, Google Scholar, Scopus and ScienceDirect to identify relevant studies investigating the effects of exercise interventions in adults with MG. Keywords were organized into three thematic groups. The full search strategy used for PubMed was as follows: (“myasthenia gravis”) AND (“exercise” OR “physical activity” OR “aerobic training” OR “resistance training” OR “rehabilitation” OR “respiratory muscle training”).

Equivalent search terms and Boolean operators were adapted for Web of Science, Scopus, ScienceDirect, and Google Scholar. Filters applied included: English language, human subjects, and publication date from 2015 to 2025. This time restriction was applied to capture the most recent evidence reflecting current clinical practices and methodological standards in exercise-based interventions for myasthenia gravis. Earlier studies were considered in the background literature to contextualize findings; however, they were not included in the systematic synthesis to maintain consistency with contemporary rehabilitation approaches. All retrieved recorders were imported into EndNote software (Version X20 for Windows 11, Thomson Reuters, New York, NY, USA) for reference management and duplication.

### 2.2. Inclusion and Exclusion Criteria

Studies were eligible for inclusion if they were original interventional investigations, including randomized or controlled clinical trials, published in English within the last ten years and enrolling adult participants with a confirmed diagnosis of MG. The ten-year timeframe was chosen to ensure inclusion of the most recent and clinically relevant evidence reflecting current practices and methodological standards in the field. To ensure clinical relevance and methodological consistency, only studies in which exercise or structured physical activity constituted the primary intervention were considered. Eligible studies were required to include a control or comparison group, report pre- and post-intervention outcomes, and describe disease severity using the Myasthenia Gravis Foundation of America (MGFA) classification. Exclusion criteria comprised observational studies, case reports, conference abstracts, non-human studies, or studies in which exercise was not the primary focus of the intervention. Studies were required to provide a clear description of the exercise intervention, including type, intensity, duration, and/or frequency, to allow meaningful comparison across interventions.

These criteria were applied to ensure that the selected evidence specifically addressed the effects of structured exercise interventions within a controlled experimental framework. The study section was guided by the population, intervention, comparison, outcome, and study design (PICOs) framework, summarized in Table 1.

### 2.3. Study Selection and Data Extraction

Database searches and duplicate studies were independently conducted by two reviewers. The same reviewers then performed study selection in two sequential stages: (a) screening of titles and abstracts, followed by (b) full-text assessment according to the predefined inclusion and exclusion criteria. Inter-reviewer consistency was ensured through predefined inclusion and exclusion criteria and standardized data extraction procedures.

Discrepancies were resolved through discussion with a third reviewer. For each included study, data were systematically extracted and recorded in a Microsoft Excel spreadsheet (Microsoft Corp., Redmond, WA, USA) including study design, sample characteristics, MGFA classification, type and duration of the exercise intervention, outcome measures, and main findings. This process enabled a structured comparison of intervention characteristics and outcomes across studies. Due to clinical and methodological heterogeneity among the included studies, a meta-analysis was not performed. Therefore, no pooled effect estimated (e.g., mean difference or standardized mean difference) was calculated. Results are presented descriptively, reporting within-group and between-group differences as described in the original studies.

No formal subgroup analyses or meta-regression were conducted due to the absence of quantitative synthesis. However, studies were grouped according to exercise modality (aerobic, resistance, combined, respiratory) to explore potential sources of clinical heterogeneity.

No restrictions based on methodological quality were applied during study selection in order to avoid selection bias.

Sensitivity analyses were not performed due to the qualitative nature of the synthesis and the limited number of controlled studies available.

### 2.4. Methodological Quality Assessment

The methodological quality of included studies was assessed using the modified 27-item version of the Downs and Black checklist [9]. This validated tool evaluates multiple domains, including reporting quality, external validity, internal validity related to bias and confounding, and statistical power. In the modified version, total scores range from 0 to 28, with higher scores indicating better methodological quality [10]. For interpretative purposes, studies were categorized as excellent (26–28), good (20–25), fair (15–19), or poor (<14). The application of this checklist allowed for a standardized assessment of study quality and supported a critical interpretation of the evidence base [11]. The certainty of the evidence for the main outcomes (functional capacity, muscle strength, respiratory function, fatigue, and quality of life) was assessed using the Grading of Recommendations Assessment, Development and Evaluation (GRADE) approach. This framework evaluates evidence across five domains: risk of bias, inconsistency, indirectness, imprecision, and publication bias. Each domain was assessed to determine potential downgrading, leading to an overall rating of certainty classified as high, moderate, low, or very low.

Publication bias and reporting bias were not formally assessed (e.g., funnel plot analysis) due to the absence of meta-analysis and the small number of included studies. However, efforts were made to minimize selection bias by conducting a comprehensive search across multiple databases.

Methodological quality was not used as an inclusion or exclusion criterion but was considered in the interpretation of the findings.

Detailed results of both the Downs and Black methodological quality assessment and the full GRADE are provided in the Supplementary Material.

## 3. Results

### 3.1. Study Selection and Overview

**Figure 1:**
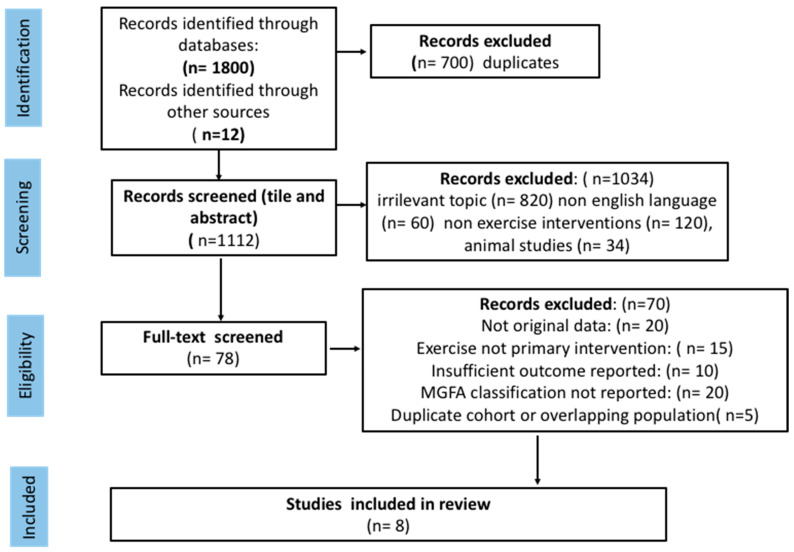
The flow diagram represents the selection process of records.

A total of 1812 records were identified across all databases before duplication and screening. After screening titles, abstracts, and full texts, eight controlled interventional studies met the inclusion criteria and were included in this qualitative synthesis. The included trials investigated aerobic exercise, respiratory muscle resistance training and combined aerobic, resistance and respiratory training programs in adults with clinically stable MG. Sample sizes ranged from small pilot cohorts to moderate-sized randomized controlled trials, with participants predominantly classified as MGFA I–III. Further details on the study selection process are presented in Figure 1.

Methodological rigor varied across studies between excellent and poor, including differences in randomization, control of confounding factors, blinding, and follow-up duration. While some trials demonstrated robust reporting and internal validity, others were limited by small sample size or less standardized intervention programs. Overall, structured exercise interventions were generally safe and well-tolerated across all exercise modalities. Improvements in functional capacity, muscle strength, and respiratory outcomes were frequently reported, although the strength of conclusions is constrained by heterogeneity in study design, sample size, and intervention programs. The certainty of evidence, assessed using the GRADE approach, ranged from very low to moderate across outcomes, with most outcomes downgraded due to study limitations, inconsistency, and imprecision. Further details on the study characteristics are presented in Table 2.

### 3.2. Aerobic Exercise Interventions

Aerobic exercise has been evaluated in several randomized and controlled trials, most commonly using walking or cycle ergometer programs. Misra et al. [16] compared a structured 30 min aerobic program with a rest condition. At 3 months, the EG showed significant improvements in QoL (*p* = 0.020) and 6 min walking distance (*p* = 0.007). No between-group differences were observed for muscle score improvement. No intervention-related adverse events were reported and compliance was >97% in both groups. The study was characterized by a controlled design and clear outcome reporting, although blinding procedures were limited. Amalina et al. [18] investigated a low-intensity aerobic cycling program in patients with MG. After 8 weeks, significant between-group differences were observed in FVC (*p* = 0.009) and FEV1 (*p* = 0.029), with higher values in the EG, while no significant differences were found for FEVR. Within-group analyses showed significant improvements in FVC and FEV1 in the EG (*p* = 0.003 and *p* = 0.029, respectively). In the CG, there was only a significant difference in FEV1 (*p* = 0.016). The study was characterized by well-defined eligibility criteria, standardized intervention protocols, and clearly reported outcomes; however, the small sample size and short intervention duration limit the generalizability and long-term interpretation of the findings. Birnbaum et al. [14] conducted a randomized controlled trial, including 43 participants (EG = 23, CG = 20), to evaluate a home-based aerobic exercise program. The EG showed a significant improvement in the 6 min walking distance test at the end of intervention (*p* = 0.01) and MG Activities of Daily Living score (*p* = 0.005). No between-group differences were found in muscle strength, pulmonary function, psychological outcomes, cytokine levels, or medication use. Exercise was associated with no increase in adverse events or MG exacerbations, and overall tolerance was comparable between groups.

### 3.3. Respiratory Muscle Resistance Training

Respiratory muscle training has been investigated in a limited number of controlled studies using diverse training modalities and study designs. Freitag et al. [15] examined long-term respiratory muscle training in patients with MG. 12 participants in the EG and 5 in the CG completed the study. Exercise significantly increased time to exhaustion in the EG at 3 and 12 months (*p* < 0.001), with no changes in the CG. EG also showed reduced perceived respiratory effort (*p* = 0.022), improved ventilatory regularity (*p* = 0.019), increased MVV15 (*p* = 0.026), improved MG symptom score (*p* < 0.001) and higher squat performance (*p* = 0.022), while no significant changes were observed in the CG. Overall, respiratory muscle training appears to be a promising adjunctive intervention for patients with MG, particularly given the potential involvement of the respiratory musculature even in clinically stable disease. However, compared with aerobic -based interventions, the existing evidence is methodologically limited. These limitations underscore the need for rigorously designed trials with standardized programs and longer follow-up to more clearly define the role of respiratory-focused training in MG rehabilitation.

### 3.4. Combined Aerobic, Resistance and Respiratory Training Programs

Combined aerobic-resistance training programs have been investigated in several controlled studies. Rahbek et al. [12] compared aerobic and resistance training in patients with mild MG. The study reported improvements in isometric muscle strength and functional performance following resistance training, while aerobic training showed modest improvements in physical function. No significant changes in VO_2_ peak were observed in either group. Between-group analyses indicated no statistically significant differences for most outcomes, except for specific functional performance measures favoring resistance training. Westerberg et al. [13] investigated the effects of supervised exercise intervention including aerobic training, resistance training and balance exercise in patients with MG. Following the intervention, significant improvements were observed in physical performance outcomes, including the 6 min walk test (*p* = 0.002) and 30 s chair stand test (*p* = 0.003). Compound motor action potential amplitudes increased significantly in the biceps and quadriceps muscles (*p* = 0.002 and *p* = 0.037, respectively). Several measures of muscular strength and body composition also showed statistically significant improvements after the training period. Chen et al. [17] evaluated respiratory muscle resistance training combined with aerobic exercise in a perioperative MG population. The intervention was associated with significantly higher postoperative vital capacity (VC, FVC, FEV1 and PEF; *p* < 0.05) and improved Activities of Daily Living score recovery at day 5 (*p* = 0.001), while no significant between-group differences were observed for 6 min walking distance (*p* > 0.05). Overall, combined training was associated with improvements in respiratory function and functional recovery; however, variability in outcome measures and study design across trials limits the robustness of the conclusions. Chang et al. [19] investigated the effects of inspiratory muscle resistance training combined with aerobic exercise in a randomized controlled trial. After 6 weeks, the EG showed significant improvements in respiratory muscle strength (*p* < 0.001), exercise capacity (*p* < 0.001), and pulmonary function (FVC and FEV1; *p* < 0.01), with significant between-group differences for most outcomes. The study demonstrated a controlled design and use of standardized outcome measures; however, limited blinding and short follow-up reduced the strength of conclusions regarding long-term effects. Additional information on the training program is summarized in Table 3.

## 4. Discussion

The role of physical activity in neuromuscular disorders (NMD) has been investigated in several studies, including our previous articles in a cohort of 149 patients affected by different NMDs, including 49 MG patients during the COVID-19 pandemic. in that context, increased sedentary behaviors and reduced physical activity were associated with a negative impact on lifestyle and functional status in patients with neuromuscular diseases (including MG) [20]. In the subsequent study, conducted three years later, preliminary observations suggested potential benefits associated with the return to a healthier lifestyle [21]. This systematic review synthesizes evidence from eight controlled interventional studies investigating the effects of structured exercise programs in adults with MG [12,13,14,15,16,17,18,19]. By focusing on controlled studies with pre and post evaluation, and clear clinical characterization, the review provides an overview of the current evidence regarding the potential impact of exercise on physical function, respiratory performance, and clinical stability in MG. However, the limited number of eligible trials, together with their methodological heterogeneity, requires cautious interpretation of the findings. Across the included studies, aerobic exercise appears to be a feasible and generally well-tolerated intervention in clinically stable patients with MG. Walking programs and low-intensity cycle ergometry were associated with improvements in functional capacity, pulmonary function, and activities of daily living, without evidence of sustained worsening of symptoms [13,14,16,18]. These findings are in line with previous literature suggesting that aerobic conditioning may help counteract physical deconditioning and reduced cardiorespiratory fitness commonly observed in MG [4,5,22]. Importantly, aerobic interventions were generally moderate or low in intensity and closely supervised, highlighting the relevance of individualized exercise prescription for ensuring safety and tolerability [1,2]. Resistance and combined aerobic-resistance programs also appear to have potential benefits, particularly in muscle strength, neuromuscular performance, and functional outcomes. Rahbek et al. [12] reported that both aerobic and resistance training could be implemented without clinical deterioration, while other studies suggested improvements in physical capacity and patient-reported outcomes [13,14]. Overall, these findings indicate that progressive resistance training, when carefully prescribed and monitored, may be feasible in selected patients with stable MG, although the available evidence remains limited [7,23,24]. Respiratory muscle training represents another relevant intervention, given the potential involvement of respiratory muscles even in patients without overt respiratory failure. Some studies suggest that inspiratory or endurance-based respiratory training may improve respiratory muscle strength and pulmonary function [15,17,19]. However, results were more heterogeneous compared with aerobic exercise, reflecting differences in training protocols, duration, and patient characteristics. Therefore, conclusions regarding its effectiveness remain preliminary and require confirmation in larger and more standardized studies [25,26]. An additional aspect emerging from this synthesis is the comparison between combined (multimodal) interventions and single-component programs. Overall, combined interventions integrating aerobic, resistance, and/or respiratory training tended to produce broader improvements across multiple domains, including functional capacity, respiratory performance, and activities of daily living. In contrast, single-component interventions, such as aerobic or respiratory muscle training alone, appeared to yield more domain-specific benefits (e.g., aerobic exercise primarily improving functional capacity and QoL, and respiratory training targeting pulmonary outcomes). However, direct comparisons between modalities remain limited, and the superiority of combined approaches cannot be definitively established due to heterogeneity in study design and outcome measures. From a clinical perspective, these findings suggest that multimodal programs may be preferable when the goal is to achieve global functional improvement, whereas targeted single-component interventions may be appropriate in the presence of specific impairments or clinical priorities.

Differences in disease stage and severity also appear to influence both the feasibility and the effectiveness of exercise interventions. Most included studies enrolled patients with mild to moderate disease (MGFA I–III), limiting the generalizability of findings to more severe cases. Patients with milder forms of MG were generally able to tolerate combined and higher-intensity interventions, including resistance training, with favorable outcomes in muscle strength and overall function. Conversely, individuals with moderate disease may benefit more from supervised, lower-intensity, and progressively adapted programs, with particular emphasis on aerobic conditioning and careful symptom monitoring. In addition, respiratory-focused interventions may be especially relevant in patients with early signs of respiratory involvement or in perioperative settings. These observations support the need for a stratified approach to exercise prescription in MG, where the type, intensity, and complexity of the intervention are tailored according to disease severity and clinical presentation. A consistent finding across the included studies is the absence of reported sustained worsening of MG symptoms or exercise-related serious adverse events [12,13,14,16]. These observations suggest that there is a growing consensus that appropriately prescribed physical exercise is likely safe in clinically stable MG [1,2,3], while inactivity and secondary deconditioning may contribute more substantially to functional decline and fatigue [1,4,27,28]. Evidence also suggests that exercise may increase β-endorphin levels and modulate receptor expression, potentially improving neuromuscular transmission and reducing fatigue and pain, although clinical confirmation in adults remains limited [28]. Integrating these findings with broader literature supports a shift away from exercise avoidance toward a rehabilitation-oriented approach in MG management. Observational studies have associated higher habitual physical activity with reduced fatigue and improved QoL [2,29,30]. In this context, structured exercise may also be beneficial in post-thymectomy rehabilitation, improving functional Recovery and reducing complications [31]. For example, propensity score-matched analysis has reported a reduction in postoperative complications and faster recovery of functional capacity in patients receiving targeted rehabilitation, supporting the inclusion of exercise-based interventions in the postoperative management. Another study demonstrated that spinal stabilization exercises are safe and effective in patients with MG, leading to improvements in trunk muscle endurance, balance, and functional performance. The randomized crossover design supports the role of targeted core exercise programs as a beneficial, non-pharmacological intervention in this population [32]. The controlled trials synthesized in this review complement these observations by showing that structured exercise interventions can induce measurable benefits without compromising disease stability. Together, these findings suggest that exercise may address both primary neuromuscular limitations and secondary consequences of inactivity, such as reduced aerobic capacity and muscle deconditioning [33,34]. Limited data in pediatric populations suggest similar benefits in pulmonary function, neuromuscular performance and QoL; however, the evidence remains scarce and further studies are required [35]. From a clinical perspective, the available evidence supports the inclusion of individualized exercise programs as an adjunct to standard medical therapy in patients with stable MG. Exercise prescriptions should be flexible, symptom-guided, and adapted to daily fluctuations in strength and fatigue [1,2,36]. A gradual approach is recommended, starting with low-intensity aerobic activities and progressing as tolerated, with the possible addition of resistance training. Careful monitoring of symptoms and the integration of rest intervals are essential to ensure safety and adherence. In addition, the applicability of exercise interventions may vary according to disease severity. Patients with mild or well-controlled MG are more likely to tolerate combined aerobic and resistance training, while those with moderate disease may benefit from supervised, lower-intensity and intermittent programs. This highlights the need for stratified and individualized exercise prescription across the clinical spectrum of MG. From a research standpoint, future studies should aim to harmonize intervention protocols and outcome measures, extend follow-up periods, and include a broader spectrum of disease severity. These efforts will be essential to refine exercise recommendations and clarify the role of physical training across the full clinical continuum of MG. Future research should also explore the use of digital technologies to support exercise programs, such as telecoaching and remote monitoring, which have shown promising results in other neurological populations and may help improve adherence, supervision, and individualized prescription in MG [37,38]. This review is limited by the small number of controlled interventional trials and the heterogeneity of exercise interventions. Sample sizes were generally modest, and follow-up durations were often short, limiting generalizability and precluding definitive conclusions regarding long-term outcomes and adherence. Methodological limitations were also observed across studies. While some trials demonstrated robust reporting, adequate control of confounding, and clearly defined outcome measures, others were limited by incomplete blinding, reduced external validity, or insufficient statistical power. This variability in methodological quality tempers the overall strength of the evidence and underscores the need for cautions interpretation.

In addition, the restriction to studies published within the last ten years may have limited the number of eligible studies and excluded earlier relevant evidence. This choice was made to focus on contemporary exercise protocols and methodological standards, but it may have reduced the comprehensiveness of the synthesis.

No formal assessment of reporting bias was conducted due to the qualitative nature of the synthesis and the limited number of included studies. Therefore, the potential impact of selective reporting cannot be excluded.

## 5. Conclusions

The findings of this systematic review suggest that structured exercise interventions may be safely implemented in adults with clinically stable MG and are potentially associated with improvements in physical and respiratory function. Across the included controlled studies, aerobic exercise, resistance training, combined programs, and respiratory muscle training were generally well tolerated and no sustained worsening of disease symptoms was reported. Although the available evidence indicates possible benefits on functional capacity, muscle strength, and pulmonary performance, these findings should be interpreted with caution due to the limited number of trials, small sample sizes, short follow-up durations, and heterogeneity of intervention. Overall, the current evidence suggests that individualized, symptom-guided exercise programs may be considered as a complementary component of MG management in carefully selected patients. Exercise prescriptions should emphasize gradual progression, adequate recovery, and clinical supervision. Future research should focus on well-powered randomized controlled trials with standardized outcome measures, longer follow-up periods, and inclusion of a broader spectrum of disease severity to strengthen the evidence base and inform clinical recommendations.

### Implications for Clinical Practice

In summary, the available evidence supports the inclusion of physical exercise as a complementary and individualized component in the management of clinically stable myasthenia gravis, with modality-specific considerations. Aerobic exercise represents the first-line approach due to the most consistent evidence of safety and its beneficial effects on functional capacity, exercise tolerance, and activities of daily living. Resistance training may be introduced in carefully selected and clinically stable patients, using progressive and closely supervised protocols aimed at improving muscle strength and neuromuscular performance without exacerbating fatigue. Combined aerobic–resistance programs appear to provide the most comprehensive functional benefits, particularly in mildly affected individuals, although they require higher levels of supervision and individualized prescription. Respiratory muscle training emerges as a valuable adjunctive intervention, especially in patients with respiratory involvement or in perioperative settings, although its evidence base remains more heterogeneous. Overall, these findings support a stratified and adaptive rehabilitation model in which exercise is progressively prescribed, symptom-guided, and integrated with pharmacological treatment to optimize functional outcomes, independence, and quality of life while maintaining disease stability.

## Figures and Tables

**Table 1:** The PICOs framework.

PICOs Component	Details
**Population**	Adults with a confirmed diagnosis of myasthenia gravis clinically stable, classified according to MGFA I–III.
**Intervention**	Structured exercise programs, including aerobic exercise; resistance training; combined aerobic-resistance programs; respiratory muscle training.
**Comparator**	Control or comparison groups, including usual care; no intervention; alternative exercise modality or lower-intensity intervention
**Outcome**	Primary outcomes: Functional capacity; Muscle strength; Respiratory function; Fatigue; Quality of life Secondary outcomes: Safety and tolerability (adverse events, disease exacerbation)
**Study Design**	Original interventional studies, including randomized controlled trials and controlled clinical trials, published in English in the last 10 years, enrolling adult patients with myasthenia gravis and including a control group. Studies were required to report pre- and post-intervention outcomes and MGFA classification. Observational studies, case reports, conference abstracts, non-human studies, and studies in which exercise was not the primary intervention were excluded.

**Table 2:** Characteristics and main outcomes of Exercise Interventions in Patients with Myasthenia Gravis.

Study (Author, Year)	Participants (n), Age (Mean ± SD, [Median/Range] MGFA Class	Intervention	Comparator	Outcomes
**Rahbek et al., 2017 [12]**	[15] (8 ATG; 7 RTG) **ATG:** 50.2 ± 21.6 **RTG:** 61.0 ± 10.7 II–III	AE + RT	Aerobic vs. resistance groups	Moderate- to high-intensity AE and RT were feasible for patients with mild MG; RT improved maximal strength (*p* < 0.05), functional capacity (*p* = 0.08), while AE led to partial improvements in physical function; no significant changes in VO_2_ peak were observed (*p* = 0.47).
**Westerberg et al., 2017 [13]**	[10] 65 ± 14 I–IIB	AE + RT + BT	No intervention	Exercise was safe and well tolerated, with no worsening of the MG-composite score or neuromuscular function. Compound motor action potential amplitudes increased in the biceps (*p* = 0.002) and quadriceps (*p* = 0.037); and both physical fitness (*p* = 0.002) and body composition showed positive changes (*p* = 0.019).
**Birnbaum et al., 2021 [14]**	[43] 45 [16–70] II–III	AE	UC	EG demonstrated significant improvements in physical function (*p* = 0.01) and Myasthenia Gravis Activities of Daily Living score (*p* = 0.005). Exercise was well tolerated, with no improvement in QoL (*p* = 0.72). No exercise-related adverse events were reported.
**Freitag et al., 2018 [15]**	[24] (EG: 18; CG: 6) **EG:** 59.8 ± 3.1; **CG:** 55.3 ± 7.3 II	RMRT	CG	EG showed significant improvements in respiratory endurance (*p* < 0.05) and physical performance (*p* = 0.022). Additionally, RMRT led to prolonged expiration, reduced respiratory rate, and subjective improvements in MG and respiratory symptoms (*p* < 0.001). No significant changes were observed in the CG (*p* > 0.05). Intervention was safe and feasible.
**Misra et al., 2021 [16]**	[38] (19 EG; 19 CG) **EG:** 59.8 ± 3.1; **CG:** 55.3 ± 7.3 II–III	AE	RC	EG had significantly improved QoL (*p* = 0.020) and 6-Minute walking distance (*p* = 0.007). No intervention-related adverse events. Both groups improved from baseline, but the effect size was greater in EG.
**Chen et al., 2023 [17]**	[80] (40 EG; 40 CG) **EG:** 39.3 ± 12.8 **CG:** 42.9 ± 9.5 I–II	AE + RMRT	SC	Postoperative VC, FVC, FEV1, and PEF were significantly higher in the EG than in the CG (*p* < 0.05). Postoperative Activities of Daily Living score was also significantly higher in the EG (*p* = 0.001). The intervention was safe, with no reported adverse events.
**Amalina et al., 2024 [18]**	[17] (9 EG; 8 CG) **EG:** 48.4 ± 5.2; **CG:** 45.7 ± 6.9 II	AE	CG	After 8 weeks, significant improvements in FVC (*p* = 0.003) and FEV1 (*p* = 0.029) in the EG. No significant change in FEVR; post-intervention FVC and FEV1 were significantly higher in the EG compared to CG (*p* = 0.009; *p* = 0.029, respectively).
**Chang et al., 2025 [19]**	[54] (EG: 26; CG: 28) **EG:** 54.9 ± 15.2; **CG:** 56.2 ± 13.4 II–III	AE + RMRT	CG	The EG demonstrated significantly greater improvements in respiratory muscle strength (*p* = 0.001), exercise capacity (*p* = 0.001), and pulmonary function (*p* = 0.01).

Legend. EG: Exercise Group; CG: Control Group; MGFA: Myasthenia Gravis Foundation of America; ATG: Aerobic Training Group; RTG: Resistance Training Group; AE: Aerobic Exercise; RT: Resistance Training; BT: Balance Training; RMRT: Respiratory Muscle Resistance Training; SC: Standard Care; RC: Rest Condition; 6MWT: 6-Minute Walk Test; ADL: Activities of Daily Living; QoL: Quality of Life; MVV: Maximal Voluntary Ventilation; FVC: Forced Vital Capacity; FEV1: Forced Expiratory Volume in the First Second; VC: Vital Capacity; VO_2_: Oxygen Consumption (peak oxygen uptake); CMAP: Compound Muscle Action Potential.

**Table 3:** Characteristics of Exercise Interventions in Patients with Myasthenia Gravis.

First Author, Year	Training Program
**Rahbek et al., 2017 [12]**	5×/week for 8 weeks **AE:** 3 sets of 10–12 min cycling on a bicycle ergometer with 3 min rest; intensity progressed from 70% to 85% of maximal heart rate over 8 weeks **RT:** Full-body program, weighted step-up, Smith bench press, leg press, pull-down, hip-flexion, lateral raises; progressed from 3 sets × 12 reps at 15 RM (week 1) to 3 sets × 8 reps at 8 RM (week 8); 90–120 s rest between sets
**Westerberg et al., 2017 [13]**	2×/week for 12 weeks **AE:** Stationary bicycle—5 min warm-up, 7× (2 min high load + 1 min low load), 5 min cool-down; **RT:** 8 resistance exercises (biceps curl, triceps pushdown, seated leg curl, cable pull-down, leg extension, cable rowing, sit-ups, leg press); 2 sets × 10RM **BT:** 1-leg standing, 1 min per leg on variable surfaces
**Freitag et al., 2018** **[15]**	5×/week for 4 weeks (IT), then 5 sessions/2 weeks for 12 months, 30 min sessions **RMRT:** Respiratory muscle exercise with portable rebreathing device; tidal volume 50–60% VC, breathing rate 25–35/min; intensity adjusted to tolerance.
**Birnbaum et al., 2021** **[14]**	3×/week for 12 weeks, 40 min sessions **AE:** Rowing ergometer, 10 min warm-up to reach individual target HR (70% HRmax), 20 min steady-state rowing at target HR, 5 min power interval phase (5 sets of 10 maximal-effort pulls at the start of each minute followed by regular-intensity strokes), and 5 min active cool-down.
**Misra et al., 2021** **[16]**	1–2 sessions/day for 12 weeks **AE:** Daily walking, progressed from 10 min/day (week 1) to 20 min/day (week 2) and 30 min/day from week 3 onward, continued for 3 months. Walking was performed at home or outdoors.
**Chen et al., 2023** **[17]**	5×/week for 5 days (pre-operative) and daily post-operative for 5 days **AE:** Treadmill and stair climbing, 2 × 45 min/day preoperatively; repetitions increased postoperatively **RMRT:** Inspiratory muscle training with 3-ball trainer, 3 × 15 min/day pre-op; deep inspiratory and abdominal pressure breathing post-op, 10–15 reps per exercise, repetitions increased daily **RT:** Limb exercises with elastic bands (shoulder, hip, knee), 10 reps × 3 sets/day post-op, repetitions increased by 5/day for 5 days
**Amalina et al., 2024** **[18]**	3×/week for 8 weeks, 30 min sessions **AE:** Cycle ergometer, 5 min warm-up, 20 min core exercise at 11–12 Borg RPE (target HR = rest HR + 30% HR reserve), 5 min cool-down
**Chang et al., 2025** **[19]**	3×/week for 8 weeks, 30-min sessions **AE:** upper limb exercises, lower limb stepping warm-up and walking training. Intensity maintained at 50–70% HRmax. 2×/day for 6 weeks, 10–15 min sessions **RMRT:** 6 sets × 5 breaths per session, inhale deeply against resistance, hold breath for 3 s, controlled exhalation; sessions include warm-up, endurance, and cool-down phases, with additional rest periods as needed to prevent hyperventilation.

Legend. EG: Exercise Group; CG: Control Group; MG: Myasthenia Gravis; AE: Aerobic Exercise; RT: Resistance Training; BT: Balance Training; RMRT: Respiratory Muscle Resistance Training; VC: vital capacity; FVC: forced vital capacity, FEV1: forced expiratory volume in the first second; PEF: peak expiratory flow rate; FEVR: forced expiratory volume ratio; ADL: activities of daily living.

## Data Availability

No new data were created or analyzed in this study.

## Supplementary Materials

The following supporting information can be downloaded at: https://www.mdpi.com/article/10.3390/healthcare14081100/s1, Table S1: Quality assessment of the included studies.
