# Supplementary material for: Physical Exercise in Myasthenia Gravis: A Systematic Review
Source: Healthcare (Basel). 2026 Apr 20;14(8):1100. doi: 10.3390/healthcare14081100 (PMC13115947; doi:10.3390/healthcare14081100)
Supplement: Supplementary file 1 [file healthcare-14-01100-s001.zip › healthcare-4175412-supplementary.pdf]

Table S1. Quality assessment of the included studies.

ARTICOLI	1	2	3	4	5	6	7	8	9	10	11	12	13	14	15	16	17	18	19	20	21	22	23	24	25	26	27	Total Score
**Rahbek et al. (2017)**	1	1	1	1	1	1	1	1	1	1	0	1	1	1	0	0	1	1	1	1	1	1	0	0	1	1	0	21
**Westerberg et al. (2017)**	1	1	0	1	1	1	1	0	1	1	0	1	1	1	0	0	1	1	1	1	0	0	0	1	0	0	0	16
**Birnbaum et al.(2021)**	1	1	0	1	1	1	1	0	1	1	0	1	1	1	0	0	1	1	1	1	0	1	0	1	0	0	1	18
**Freitag et al. (2018)**	1	1	1	1	0	1	1	1	1	1	0	0	0	0	0	1	1	0	1	0	0	0	1	0	0	0	0	13
**Misra et al. (2021)**	1	1	1	1	1	1	1	1	1	1	0	0	1	0	0	1	1	1	1	1	1	0	1	0	1	1	1	20
**Chen et al. (2023)**	1	1	1	1	1	1	1	1	1	1	0	0	1	0	0	1	1	1	1	1	1	0	1	0	1	1	1	21
**Amalina et al. (2024)**	1	1	1	1	1	1	1	1	1	0	1	1	0	0	0	1	1	1	1	1	1	1	1	0	1	1	1	22
**Chang et al. (2025)**	1	1	1	1	2	1	1	1	1	1	1	1	0	0	1	1	1	1	1	1	1	1	1	1	1	1	1	26

U, unable to determine; 1, yes; 0, no. For item 5: 0, no; 1, partially; 2, yes.

Downs and Black checklist items: Reporting [(1) Is the hypothesis/aim/objective of the study clearly described?; (2) Are the main outcomes to be measured clearly described in the Introduction or Methods section?; (3) Are the characteristics of the patients included in the study clearly described?; (4) Are the interventions of interest clearly described?; (5) Are the distributions of principal confounders in each group of subjects to be compared clearly described?; (6) Are the main findings of the study clearly described?; (7) Does the study provide estimates of the random variability in the data for the main outcomes?; (8) Have all important adverse events that may be a consequence of the intervention been reported?; (9) Have the characteristics of patients lost to follow-up been described?; (10) Have actual probability values been reported (e.g., 0.035 rather than <0.05) for the main outcomes except where the probability value is less than 0.001?]; External validity [(11) Were the subjects asked to participate in the study representative of the entire population from which they were recruited?; (12) Were those subjects who were prepared to participate representative of the entire population from which they were recruited?; (13) Were the staff, places, and facilities where the patients were treated, representative of the treatment the majority of patients receive?]; Internal validity – bias [(14) Was an attempt made to blind study subjects to the intervention they have received?; (15) Was an attempt made to blind those measuring the main outcomes of the intervention?; (16) If any of the results of the study were based on "data dredging," was this made clear?; (17) In trials and cohort studies, do the analyses adjust for different lengths of follow-up of patients, or in case-control studies, is the time period between the intervention and outcome the same for cases and controls?; (18) Were the statistical tests used to assess the main outcomes appropriate?; (19) Was compliance with the intervention/s reliable?; (20) Were the main outcome measures used accurate (valid and reliable)?]; Internal validity – confounding (selection bias) [(21) Were the patients in different intervention groups (trials and cohort studies) or were the cases and controls (case-control studies) recruited from the same population?; (22) Were study subjects in different intervention groups (trials and cohort studies) or were the cases and controls (case-control studies) recruited over the same period of time?; (23) Were study subjects randomized to intervention groups?; (24) Was the randomized intervention assignment concealed from both patients and health care staff until recruitment was complete and irrevocable?; (25) Was there adequate adjustment for confounding in the analyses from which the main findings were drawn?; (26) Were losses of patients to follow-up taken into account?]; (27) Power: Did the study have sufficient power to detect a clinically important effect where the probability value for a difference being due to chance is less than 5%?
